# Family with systemic arterial hypertension: epidemiological profile

**DOI:** 10.17843/rpmesp.2022.392.11211

**Published:** 2022-06-30

**Authors:** Enrique Villarreal Ríos, Argenes Huato Solorio, Liliana Galicia Rodríguez, Verónica Escorcia Reyes, Emma Rosa Vargas Daza, Erasto Carballo Santander

**Affiliations:** 1 Unidad de Investigación Epidemiológica y en Servicios de Salud Querétaro, Instituto Mexicano del Seguro Social, Querétaro, México. Instituto Mexicano del Seguro Social Unidad de Investigación Epidemiológica y en Servicios de Salud Querétaro Instituto Mexicano del Seguro Social Querétaro Mexico; 2 Residencia de Medicina Familiar n.° 6, Unidad de Medicina Familiar n.° 6 San Juan del Río Querétaro, Instituto Mexicano del Seguro Social, Querétaro, México. Instituto Mexicano del Seguro Social Residencia de Medicina Familiar n.° 6 Unidad de Medicina Familiar n.° 6 San Juan del Río Querétaro Instituto Mexicano del Seguro Social Querétaro Mexico; 3 Coordinación de Educación e Investigación, Unidad de Medicina Familiar n.° 6 San Juan del Río Querétaro, Instituto Mexicano del Seguro Social, Querétaro, México. Instituto Mexicano del Seguro Social Coordinación de Educación e Investigación Unidad de Medicina Familiar n.° 6 San Juan del Río Querétaro Instituto Mexicano del Seguro Social Querétaro Mexico; 4 Coordinación de Educación e Investigación, Unidad de Medicina Familiar n.° 7 San Juan del Río Querétaro, Instituto Mexicano del Seguro Social, Querétaro, México. Instituto Mexicano del Seguro Social Coordinación de Educación e Investigación Unidad de Medicina Familiar n.° 7 San Juan del Río Querétaro Instituto Mexicano del Seguro Social Querétaro Mexico

**Keywords:** Arterial hypertension, Family, Epidemiological profile, Role, Health Services, Epidemiology (source: MeSH NLM).

## Abstract

The aim of this study was to determine the epidemiological profile of the family with systemic arterial hypertension. A descriptive cross-sectional study was carried out in 268 families with this disease, the epidemiological profile included seven dimensions, sociodemographic, economic, family functionality, life cycle, family roles, health and use of services. The mean age of the families was 49.09 (SD: 15.57) years; 47.0% of the families had paid economic activity, 65.0% were functional, 52.4% were in the retirement and death stages, 43.1% presented obesity, in 50.0% the predominant role of the hypertensive patient was assumed by the mother, and the average annual number of family medicine consultations was 10.37 (SD: 4.31). The family with arterial hypertension is functional, although most of them are in the stage of retirement and death.

## INTRODUCTION

The family defines the behavior and health decisions of its members; it is the foundation of culture, values and social norms, and it is also the basic unit of social organization that is accessible for preventive interventions, health promotion and treatment ^1^ ^-^ ^3^. The presence of acute, chronic or terminal illness in any member of the family has repercussions on the family nucleus, and in order to adapt to this situation, mechanisms that modify interaction, balance and function are set in motion ^4^ ^,^ ^5^.

In this scenario, the family takes shape and acquires particular characteristics that define and differentiate it ^1^ ^,^ ^2^ ^,^ ^6^ ^,^ ^7^. Particularly regarding systemic arterial hypertension as a family disease ^8^ ^,^ ^9^, the family develops a structural and behavioral profile ^1^ ^,^ ^3^.

Identifying the epidemiological profile provides information that allows the design of specific public policies with a high probability of being effective. In the context of family medicine, approaching the family with systemic arterial hypertension implies going beyond the individual level and placing oneself in the integral perspective of the family environment, a condition that places the family as the unit of analysis ^2^ ^,^ ^10^ ^,^ ^11^.

Given this scenario, our study aimed to determine the epidemiological profile of the family with systemic arterial hypertension who attended medical controls in a Mexican social security institution during the year 2021, considering the family as the unit of analysis.

**KEY MESSAGES**

Motivation for the study: To assume the family as the unit of analysis in order to move from the individual to the integral level and to define the characteristics of the family with systemic arterial hypertension.

Main findings: The average number of members was 3.05 (SD: 1.60), the average age was 49.09 (SD: 15.57) years; 52.4% of the families are in the retirement and death phase; 67.1% have permanent income; one out of three families are dysfunctional and the role of the patient with systemic arterial hypertension corresponds to the mother in 50% of the families.

Implications: These results will allow us to identify areas of opportunity for the implementation of public policies aimed at hypertension control.

## THE STUDY

### Design, population and unit of analysis

Descriptive cross-sectional study of families who attended a medical unit of the Mexican Social Security Institute in the city of San Juan del Río, Querétaro, Mexico, for hypertension control. The unit of analysis was the family with arterial hypertension, defined as the family with one or more members with arterial hypertension more than one year after diagnosis. Families with secondary systemic arterial hypertension, families with gestational hypertension/preeclampsia, and families with hypertension and diabetes *mellitus* together were excluded.

### Sample size

We determined the sample size with the averaging formula for infinite population, assuming that the mean age of the family with arterial hypertension was 59 years (µ=59) (12), 95% confidence level for a rejection zone (Zα=1.64), standard deviation of 5.9 (s=5.9), and a margin of error of 0.59 (d=0.59). The calculated sample size was of 268 (n=268) families with arterial hypertension.

We used the non-random sampling technique for consecutive cases, using the families who went for follow-up with the family physician as a sampling frame. To this end, the researcher went to the waiting room of the medical unit every day until the sample was complete, identifying the families with arterial hypertension in each office, and inviting them to participate. After authorizing their participation and obtaining their informed consent, participants were taken to the office for the interview.

### Variables

Seven dimensions of the epidemiological profile of the family with arterial hypertension were evaluated through the variables identified below: ^13^.

### Socio-demographic profile

Average number of family members; average age of the family (age of all family members among the total number of family members); sex of the family (five types of families were defined: families composed only by men; families with male predominance; families with equal numbers of men and women; families with female predominance, and families composed only by women); educational level of the family (highest school grade of any of the members); family religion (religion practiced by the family members) and marital status (of the members with arterial hypertension).

### Economic profile

Family socioeconomic level determined by the questionnaire of the Mexican Association of Market Intelligence and Opinion Agencies (AMAI) which defines seven categories, A/B, C+, C, C-, C+, D+, D and E based on the following five criteria: level of schooling of the head of household; permanent availability of Internet in the home; percentage of economic income on food, education and clothing (A/B is the highest level and E the lowest level) ^14^ and source of family income (paid labor activity or retirement income).

### Family functionality profile

Determined by the questionnaire for the assessment of family functionality (Family APGAR, which defines four categories: functional family, mild dysfunction, moderate dysfunction, and severe dysfunction, which in turn is assessed by five components: adaptation, participation, resource gradient, affectivity and resolve) ^15^.

### Life cycle stage profile of the family member

Determined by Geyman’s life cycle classification (defines five stages: marriage, expansion, dispersion, independence, and retirement and death. Which were defined according to the integration, growth and dissolution of the family group) ^16^.

### Family role profile

Determined by the classification of traditional roles: mother, father, sibling, grandparent, nephew, cousin, other (we identified the role of the member with arterial hypertension with the longest evolution time).

### Health profile

Time of hypertension evolution (the family member with the longest evolution); average number of members diagnosed with hypertension; comorbidities in the family (one or more members with obesity, epilepsy, migraine, chronic obstructive pulmonary disease, thyroid disease, heart disease, lupus/thrombophilia, cancer, cirrhosis/liver disease, cerebral vascular event or COVID-19 sequelae), and habits in the family (smoking or alcoholism in one or more of the family members).

### Health services use profile

Average number of health care visits per year per family and average number of visits per year per family adjusted for family members (dentistry, family planning, social work, family medicine consultation for reasons other than hypertension, preventive medicine, nutrition, laboratory, radiology, emergencies, maternal-child nursing, and family medicine consultation for hypertension).

### Statistical analysis

The statistical analysis plan included the Kolmogorov-Smirnov normality test. The mean and standard deviation (SD) were used for continuous and discrete variables and frequencies and percentages were used for categorical variables. We used the SPSS statistical program.

### Ethical considerations

The information was handled with confidentiality and was used exclusively for achieving the objectives of the study. The project was authorized by the Ethics and Research Committee of the Hospital General Regional 1 of the Instituto Mexicano del Seguro Social de Querétaro México, under number 2021-2201-049.

## FINDINGS

### Sociodemographic profile

The average number of family members was 3.05 (SD: 1.60), the average age was 49.09 (SD: 15.57) years; the most frequent level of education was high school in 33.1% of the families; the most frequent marital status of the member with arterial hypertension was married with 31.7%; exclusive Catholic religion was found in 86.0% of the families, and 45.9% of the families had an equal number of men and women (Table 1).

**Table 1:** Sociodemographic profile of the family with systemic arterial hypertension (n=268 families with arterial hypertension).

Characteristic	n	(%)
Sex of the family		
Family of men only	5	1.9
Family with male predominance	55	20.5
Family with equal number of men and women	123	45.9
Family with female predominance	64	23.9
Family of women only	21	7.8
Educational level of the family ^a^		
Bachelor Degree	65	24.3
Preparatory	89	33.1
Secondary	68	25.4
Primary	33	12.3
No schooling	13	4.9
Family religion		
Exclusively Catholic	231	86.0
Other	37	14.0
Marital status of the member with arterial hypertension		
Married	85	31.7
Widower	73	27.3
Single	31	11.6
Married and single	26	9.7
Free union	26	9.7
Divorced	17	6.3
Other	10	37

a Highest level of one of the family members

### Economic profile

The predominant socioeconomic levels in the family with arterial hypertension were “C-” and “D” with 22.0% each, and 47.0% of the families had a permanent paid job (Table 2).

**Table 2:** Economic profile of the family with systemic arterial hypertension (n=268 families with arterial hypertension).

Characteristics	n	%
Socioeconomic level of the family ^a^		
A/B	16	6.0
C	50	18.7
C-	59	22.0
C+	34	12.7
D	59	22.0
D+	36	13.4
E	14	5.2
Source of family income		
Paid job	126	47.0
Retired	54	20.2
Other source of income	88	32.8

a Questionnaire of the Mexican Association of Market and Opinion Intelligence Agencies (AMAI)

### Family functionality profile

Sixty-five percent were functional families (Table 3).

**Table 3:** Family function profile, family role profile, and family cycle stage profile (n=268 families with arterial hypertension).

Condition	n	(%)
Family functionality profile		
Functional family	174	65.0
Mild dysfunction	59	22.0
Moderate dysfunction	15	5.6
Severe dysfunction	20	7.4
Family life cycle stage profile		
Marriage	8	2.9
Expansion	15	5.6
Dispersion	24	8.9
Independence	81	30.2
Retirement and death	140	52.4
Profile of the family role of the member with hypertension ^a^		
Mother	134	50.0
Father	88	32.8
Another member	13	4.9
Mother-Father ^b^	13	4.9
Child	9	3.4
Other	11	4.0
Health profile		
Comorbidities in the family ^c^		
Obesity	116	43.2
Cardiopathy	21	7.8
Thyroid disease	18	6.7
Migraine	14	5.2
Chronic kidney disease	13	4.8
COPD	12	4.4
Cancer	11	4.1
Cerebral vascular event	5	1.8
Other	6	2.2
Habits		
Smoking	64	23.9
Alcoholism	100	37.2

a The role of the member with arterial hypertension with the longest evolution time.

b The mother and father had the same time of evolution with arterial hypertension.

c Diabetes mellitus was not included because it was an exclusion criterion.

### Family life cycle stage profile

Most families (52.4%) were at the retirement and death stage of the life cycle (Table 3).

### Profile of family roles

The mother assumed the predominant role of the patient with arterial hypertension in 50.0% (Table 3).

### Health profile

The mean number of years of hypertension diagnosis in the family was 11.65 years (SD: 10.05); the mean number of members with hypertension in the family was 1.28 (SD: 0.84). Obesity was found in 43.1% of the families and heart disease in 7.8%. Alcoholism was found in 23.9% of the families (Table 3).

### Service use profile

The average annual number of family medicine consultations per family for reasons other than hypertension was 3.08 (SD: 4.63); the average annual number of family medicine consultations per family adjusted for number of family members was 1.09 (SD: 1.64). The average annual number of family medicine consultations for control of arterial hypertension was 12.74 (SD: 6.06) and was 10.37 (SD: 4.31) when adjusted for the number of patients with arterial hypertension per family (Table 4).

**Table 4:** Profile of annual use of health services and health profile of the family with arterial hypertension (n=268 families with arterial hypertension).

Type of service	Mean	SD
Consultations per year per family		
Maternal and child nursing	0.01	0.18
Laboratory	2.08	1.94
Family Medicine (not arterial hypertension)	3.08	4.63
Preventive medicine	1.66	1.90
Nutrition	0.85	1.14
Odontology	1.29	2.19
Family planning	0.20	1.09
Imaging	0.73	0.81
Social work	0.97	0.99
Urgencies	0.67	1.29
Family Medicine (for arterial hypertension)	12.74	6.06
Consultations per year per family adjusted by family members		
Maternal and child nursing	0.003	0.045
Laboratory	0.80	0.84
Family Medicine (not arterial hypertension)	1.09	1.64
Preventive medicine	0.61	0.77
Nutrition	0.33	0.50
Odontology	0.45	0.75
Family planning	0.08	0.46
Imaging	0.28	0.34
Social work	0.36	0.37
Urgencies	0.26	0.57
Family Medicine (for arterial hypertension) ^a^	10.37	4.31

SD: standard deviation

a Adjusted for the number of members with high blood pressure in the family.

## DISCUSSION

The average number of family members with arterial hypertension was 3.05; most were in the retirement and death stage and were functional families, the average number of years with hypertension was 11.65 and the average number of members with hypertension was 1.28. Determining these characteristics from the perspective of the family allowed us to identify the context in which systemic arterial hypertension can be treated comprehensively ^8^ ^,^ ^11^.

Arterial hypertension is a condition that usually appears after the fifth decade of life; however, when approached from the perspective of the family, it may appear after the fourth decade. This is probably due to the fact that the age of the children is included when calculating the age of the family, which necessarily decreases the average age; this would be a characteristic of the family with arterial hypertension.

Education is part of the non-drug treatment of arterial hypertension ^17^, but in order to achieve the objective, the contents should be designed according to the characteristics of the population. The families with arterial hypertension mostly have secondary education, which should be taken into account when defining the educational contents and didactic techniques, thus integrating groups of families with hypertension by educational level, a scenario that will probably be more effective.

We found that most families were functional, a characteristic that may favor the control of arterial hypertension. This is because, in theory, in a functional family the members seek, recognize, and adopt individual needs, thus facilitating the achievement of common objectives; however, this hypothesis should be tested in subsequent studies ^1^ ^,^ ^9^.

The stage of the family life cycle in eight out of ten families was that of independence and retirement, which means that the children have begun to leave the family nucleus to build another family. The analysis of this information along with the age of the family suggests that the studied families began integration as a family at an early age, so it will be necessary to compare this characteristic with that of the family with diabetes or the family with any other condition ^1^ ^,^ ^5^.

Our results show that participants constantly used health services for the monitoring of arterial hypertension; this has been previously described in the literature for individuals as a bigger effect when compared with other conditions. Although the literature mainly evaluates individuals and, considering that the purpose of our study is to evaluate the family with hypertension, individual behavior could be extrapolated to the family level.

However, we also found that the use of preventive services was low, which is far from ideal. In particular, prevention activities regarding arterial hypertension are fundamental to prevent chronic complications, which represents an area of opportunity to propose health programs ^12^.

Obesity in individuals is a serious public health problem, it is not different for families with arterial hypertension. Nearly half of the families presented obesity, a prevalence lower than that reported for the Mexican population ^18^, probably due to the grouping of several obese individuals in the same family nucleus. 

The high prevalence of obesity suggests the presence of inadequate nutritional and physical practices that favor the presence of chronic complications typical of arterial hypertension; however, the prevalence of chronic complications in the family with hypertension is lower than that reported for the general population, most likely because the unit of analysis is the family group ^19^. This scenario should be interpreted with caution when comparing prevalence between individuals and families.

One limitation of this study was the lack of both, blood pressure measurement, and the type of treatment. Furthermore, it is important to mention that the studied population corresponds to a medium-sized locality, which may imply that the profile may not necessarily be the same for families with hypertension living in large cities; this comparison could be explored by similar studies. Finally, the implications of nonprobability sampling should be mentioned too, including the limitation of extrapolating data to other populations.

In conclusion, the family with arterial hypertension is functional, although most of them are in the stage of retirement and death. Identifying the epidemiological profile of the family with systemic arterial hypertension allows us to recognize areas of opportunity for the implementation of public policies aimed at hypertension control.
