# Supplementary material for: Family with systemic arterial hypertension: epidemiological profile
Source: Rev Peru Med Exp Salud Publica. 2022 Jun 30;39(2):221–6. doi: 10.17843/rpmesp.2022.392.11211 (PMC11397663; doi:10.17843/rpmesp.2022.392.11211)
Supplement: Translation

# Translation: Familia con hipertensión arterial sistémica: perfil epidemiológico

**Authors:** Enrique Villarreal Ríos, Argenes Huato Solorio, Liliana Galicia Rodríguez, Verónica Escorcia Reyes, Emma Rosa Vargas Daza, Erasto Carballo Santander

**Affiliations:** 1 Unidad de Investigación Epidemiológica y en Servicios de Salud Querétaro, Instituto Mexicano del Seguro Social, Querétaro, Mexico.; 2 Residencia de Medicina Familiar n.° 6, Unidad de Medicina Familiar n.° 6 San Juan del Río Querétaro, Instituto Mexicano del Seguro Social, Querétaro, Mexico.; 3 Coordinación de Educación e Investigación, Unidad de Medicina Familiar n.° 6 San Juan del Río Querétaro, Instituto Mexicano del Seguro Social, Querétaro, Mexico.; 4 Coordinación de Educación e Investigación, Unidad de Medicina Familiar n.° 7 San Juan del Río Querétaro, Instituto Mexicano del Seguro Social, Querétaro, Mexico.

**Keywords:** Hipertensión arterial, Familia, Perfil Epidemiológico, Rol, Servicios de Salud, Epidemiología

## Abstract

El objetivo fue determinar el perfil epidemiológico de grupos familiares con hipertensión arterial sistémica. Se realizó un estudio transversal descriptivo en 268 familias con esta enfermedad, el perfil epidemiológico incluyó siete dimensiones: sociodemográfico, económico, funcionalidad familiar, ciclo de vida, roles familiares, salud y uso de servicios. La edad promedio de las familias fue de 49,09 (DE: 15,57) años. El 47,0% de las familias tuvieron actividad económica remunerada; el 65,0% son funcionales; en el 52,4% predominó la etapa de jubilación y muerte; en el 50,0% la madre asumió el rol predominante del paciente con hipertensión; el 43,1% de las familias presentaron obesidad y el promedio anual de consultas de medicina familiar fue de 10,37 (DE: 4,31). La familia con hipertensión arterial es funcional, no obstante la mayoría se encuentra etapa de jubilación y muerte.

## INTRODUCCIÓN

La familia define el comportamiento y las decisiones en salud de sus integrantes, en ella se originan cultura, valores y normas sociales, además es la unidad básica de organización social accesible a las intervenciones preventivas, de promoción y de tratamiento en salud ^1^ ^-^ ^3^. La presencia de enfermedad aguda, crónica o terminal en algún integrante de la familia repercute en el núcleo familiar, y para lograr adaptarse a esta situación se ponen en marcha mecanismos que modifican la interacción, el equilibrio y la función ^4^ ^,^ ^5^.

Ante ese escenario la familia se configura y adquiere características particulares que la definen y distinguen^ (^ ^1^ ^,^ ^2^ ^,^ ^6^ ^,^ ^7^. Específicamente ante la presencia de hipertensión arterial sistémica como enfermedad familiar ^8^ ^,^ ^9^, la familia desarrolla un perfil estructural y de comportamiento ^1^ ^,^ ^3^.

Identificar el perfil epidemiológico proporciona información que permite diseñar políticas públicas específicas con alta probabilidad de efectividad. En el contexto de la medicina familiar, abordar a la familia con hipertensión arterial sistémica implica superar el plano individual y ubicarse en la perspectiva integral del entorno familiar, condición que sitúa a la familia como unidad de análisis ^2^ ^,^ ^10^ ^,^ ^11^.

Ante este panorama, el objetivo del estudio fue determinar el perfil epidemiológico de la familia con hipertensión arterial sistémica que acudió a control médico en una institución de seguridad social de México durante el año 2021, considerando como unidad de análisis a la familia.

**MENSAJE CLAVE**

Motivación para realizar el estudio: asumir a la familia como unidad de análisis para transitar del plano individual al integral y definir las características de la familia con hipertensión arterial sistémica.

Principales hallazgos: el promedio de integrantes fue 3,05 (DE: 1,60), la edad promedio fue 49,09 (DE: 15,57) años; el 52,4% de las familias se encuentra en la fase de retiro y muerte; el 67,1% tiene ingresos permanentes; la disfunción familiar está presente en uno de cada tres familias y el rol del paciente con hipertensión arterial sistémica corresponde a la madre en el 50% de las familias.

Implicancias: estos resultados permitiran identificar áreas de oportunidad para la implementación de políticas públicas orientadas al control de la hipertensión.

## EL ESTUDIO

### Diseño, población y unidad de análisis

Estudio transversal descriptivo en familias que acudieron a control de hipertensión arterial a una unidad médica del Instituto Mexicano del Seguro Social de la ciudad de San Juan del Río en Querétaro, México. Se definió como unidad de análisis a la familia con hipertensión arterial, entendida como aquella familia con uno o más integrantes con hipertensión arterial de más de un año de diagnóstico. Fueron excluidas familias con hipertensión arterial sistémica secundaria, familias con hipertensión gestacional/preeclampsia, así como familias con hipertensión y diabetes *mellitus* en forma conjunta.

### Tamaño de muestra

El tamaño de muestra se obtuvo con la fórmula de promedios para población infinita, asumiendo que la edad promedio de la familia con hipertensión arterial fue 59 años (µ=59) ^12^, nivel de confianza de 95% para una zona de rechazo (Zα=1,64), desviación estándar de 5,9 (s=5,9) y margen de error de 0,59 (d=0,59). El tamaño de muestra calculado fue 268 (n=268) familias con hipertensión arterial.

Se utilizó la técnica muestral no aleatoria por casos consecutivos, empleando como marco muestral a las familias que acudieron a control con el médico familiar, para ello el investigador se presentó todos los días en la sala de espera de la unidad médica hasta completar la muestra, identificó en cada consultorio a las familias con hipertensión arterial y las invitó a participar. Luego de autorizar su participación, previo consentimiento informado, se les condujo al consultorio para realizar la entrevista.

### Variables

El perfil epidemiológico de la familia con hipertensión arterial estuvo conformado por siete dimensiones evaluadas a través de las variables que se identifican a continuación: ^13^

*Perfil sociodemográfico*

Promedio de integrantes de la familia; edad promedio de la familia (edad de todos los integrantes de la familia entre el total de integrantes); sexo familiar (se definieron cinco tipos de familias: familias integradas solo por hombres; familias con predominio de hombre; familias con igual número de hombres y mujeres; familias con predominio de mujeres, y familias integradas solo por mujeres); escolaridad de la familia (grado escolar más alto de uno de los integrantes); religión de familia (culto religioso practicado por los integrantes de la familia) y estado civil (de los integrantes con hipertensión arterial).

*Perfil económico*

Nivel socioeconómico familiar determinado por el cuestionario de la Asociación Mexicana de Agencias de Inteligencia de Mercado y Opinión (AMAI) que define siete categorías, A/B, C+, C, C-, D+, D y E en función de los siguientes cinco criterios: nivel de escolaridad del jefe de familia; disponibilidad permanente de Internet en el hogar; porcentaje del ingreso económico en alimentación, educación y vestido (A/B es el nivel más alto y E el nivel más bajo) ^14^ ^) ^y origen del ingreso económico de la familia (actividad laboral remunerada o ingreso por jubilación).

*Perfil de funcionalidad familiar*

Determinado por el cuestionario para la evaluación de la funcionalidad en la familia (APGAR Familiar, que define cuatro categorías: familia funcional, disfunción leve, disfunción moderada, y disfunción severa, que se evalúa, a su vez, mediante cinco componentes: adaptación, participación, gradiente de recursos, afectividad y capacidad resolutiva) ^15^.

*Perfil de etapa del ciclo vital de la familiar*

Determinado por la clasificación del ciclo vital de Geyman (define cinco etapas: matrimonio, expansión, dispersión, independencia, y retiro y muerte. La definición se realizó en función de la integración, crecimiento y disolución del grupo familiar) ^16^.

*Perfil de roles familiares*

Determinado por la clasificación de los roles tradicionales: madre, padre, hermano, abuelo, sobrino, primo, otro (se identificó el rol del miembro con hipertensión arterial con mayor tiempo de evolución).

*Perfil de salud*

Tiempo de evolución de la hipertensión (el integrante de la familia con más tiempo de evolución); promedio de cantidad de integrantes con diagnóstico de hipertensión arterial; comorbilidades en la familia (presencia de uno o más miembros con obesidad, epilepsia, migraña, enfermedad pulmonar obstructiva crónica, enfermedad tiroidea, cardiopatía, lupus/trombofilia, cáncer, cirrosis/enfermedad hepática, evento vascular cerebral o secuelas COVID-19), y hábitos en la familia (tabaquismo o alcoholismo en uno o más de los integrantes de la familia).

*Perfil de uso de servicios de salud*

Promedio de atenciones por servicios de salud al año por familia y promedio de atenciones al año por familia ajustado por integrantes de la familia (odontología, planificación familiar, trabajo social, consulta medicina familiar -motivo diferente a la hipertensión arterial-, medicina preventiva, nutrición, laboratorio, radiología, urgencias, enfermera materno infantil y consulta de medicina familiar por hipertensión arterial).

### Análisis estadístico

El plan de análisis estadístico incluyó prueba de normalidad de Kolmogórov-Smirnov. Para las variables continuas y discretas se usó el promedio y la desviación estándar (DE) y para las variables categóricas se usó las frecuencias y porcentajes. Se utilizo el programa estadístico SPSS.

### Consideraciones éticas

La información se manejó con confidencialidad y se utilizó exclusivamente para el cumplimiento de los objetivos del estudio. El proyecto fue autorizado por el Comité de Ética e Investigación del Hospital General Regional 1 del Instituto Mexicano del Seguro Social de Querétaro México, con el número 2021-2201-049.

## HALLAZGOS

### Perfil sociodemográfico

El promedio de integrantes de la familia fue 3,05 (DE: 1,60), la edad promedio fue 49,09 (DE: 15,57) años; la escolaridad más frecuente fue preparatoria en el 33,1% de las familias; el estado civil más frecuente del integrante con hipertensión arterial fue casado con 31,7%; la religión católica exclusiva estuvo presente en el 86,0% de las familias, y se identificó igual número de hombres y mujeres en el 45,9% de las familias (Tabla 1).

**Tabla 1:** Perfil sociodemográfico de la familia con hipertensión arterial sistémica (n=268 familias con hipertensión arterial).

Característica	n	(%)
Sexo de la familia		
Familia solo de hombres	5	1,9
Familia con predominio de hombres	55	20,5
Familia con igual número de hombres y mujeres	123	45,9
Familia con predominio de mujeres	64	23,9
Familia solo de mujeres	21	7,8
Escolaridad de la familia ^a^		
Licenciatura	65	24,3
Preparatoria	89	33,1
Secundaria	68	25,4
Primaria	33	12,3
Sin escolaridad	13	4,9
Religión de la familia		
Católica exclusiva	231	86,0
Otra	37	14,0
Estado civil del integrante con hipertensión arterial		
Casado	85	31,7
Viudo	73	27,3
Soltero	31	11,6
Casado y soltero	26	9,7
Unión libre	26	9,7
Divorciado	17	6,3
Otro	10	3,7

a Nivel más alto de uno de los miembros de la familia

### Perfil económico

Los niveles socioeconómicos predominantes en la familia con hipertensión arterial fueron «C-» y «D» con 22,0% cada uno y el 47,0% de las familias tuvieron actividad laboral remunerada permanente (Tabla 2).

**Tabla 2:** Perfil económico de la familia con hipertensión arterial sistémica (n=268 familias con hipertensión arterial)

Características	n	%
Nivel socioeconómico de la familia ^a^		
A/B	16	6,0
C	50	18,7
C-	59	22,0
C+	34	12,7
D	59	22,0
D+	36	13,4
E	14	5,2
Origen del ingreso económico de la familia		
Actividad laboral remunerada	126	47,0
Jubilado	54	20,2
Otra fuente de ingreso	88	32,8

a Cuestionario de Asociación Mexicana de Agencias de Inteligencia de Mercado y Opinión (AMAI)

### Perfil de funcionalidad familiar

El 65,0% fueron familias funcionales (Tabla 3).

### Perfil de etapa del ciclo vital familiar

La etapa del ciclo vital familiar predominante fue la de jubilación y muerte con 52,4% (Tabla 3).

**Tabla 3:** Perfil de función familiar, perfil de rol familiar y perfil de la etapa del ciclo familiar (n=268 familias con hipertensión arterial).

Condición	n	(%)
Perfil de funcionalidad familiar		
Familia funcional	174	65,0
Disfunción leve	59	22,0
Disfunción moderada	15	5,6
Disfunción severa	20	7,4
Perfil de la etapa del ciclo vital familiar		
Matrimonio	8	2,9
Expansión	15	5,6
Dispersión	24	8,9
Independencia	81	30,2
Jubilación y muerte	140	52,4
Perfil del rol familiar del miembro con hipertensión ^a^		
Madre	134	50,0
Padre	88	32,8
Otro integrante	13	4,9
Madre-Padre ^b^	13	4,9
Hijo	9	3,4
Otro	11	4,0
Perfil de salud		
Comorbilidades en la familia ^c^		
Obesidad	116	43,2
Cardiopatía	21	7,8
Enfermedad tiroidea	18	6,7
Migraña	14	5,2
Enfermedad renal crónica	13	4,8
EPOC	12	4,4
Cáncer	11	4,1
Evento vascular cerebral	5	1,8
Otros	6	2,2
Hábitos		
Tabaquismo	64	23,9
Alcoholismo	100	37,2

a Se identificó el rol del miembro con hipertensión arterial con mayor tiempo de evolución.

b La madre y el padre tienen el mismo tiempo de evolución con hipertensión arterial.

c No se incluye diabetes mellitus debido a que fue un criterio de exclusión.

### Perfil de roles familiares

La madre asumió el rol predominante del paciente con hipertensión arterial en el 50,0% (Tabla 3).

### Perfil de salud

El promedio de años de diagnóstico de hipertensión arterial en la familia fue de 11,65 años (DE: 10,05); el promedio de integrantes con hipertensión en la familia fue 1,28 (DE: 0,84). El 43,1% de las familias presentaron obesidad y el 7,8% presentaron cardiopatía. El hábito del alcoholismo estuvo presente en el 23,9% de las familias (Tabla 3).

### Perfil de uso de servicios

El promedio anual del número de consultas de medicina familiar por familia por motivo diferente a la hipertensión fue de 3,08 (DE: 4,63); el promedio anual de consultas de medicina familiar por familia ajustado por número de integrantes de la familia fue 1,09 (DE: 1,64). El promedio anual de consultas de medicina familiar para control de hipertensión arterial fue de 12,74 (DE: 6,06) y cuando se ajusta por número de pacientes con hipertensión arterial por familia fue de 10,37 (DE: 4,31) (Tabla 4).

**Tabla 4:** Perfil de uso anual de servicios de salud y perfil de salud de la familia con hipertensión arterial (n=268 familias con hipertensión arterial)

Tipo de servicio	Promedio	DE
Consultas al año por familia		
Enfermera materno infantil	0,01	0,18
Laboratorio	2,08	1,94
Medicina familiar (no hipertensión arterial)	3,08	4,63
Medicina preventiva	1,66	1,90
Nutrición	0,85	1,14
Odontología	1,29	2,19
Planificación familiar	0,20	1,09
Imagenología	0,73	0,81
Trabajo social	0,97	0,99
Urgencias	0,67	1,29
Medicina familiar (por hipertensión arterial)	12,74	6,06
Consultas al año por familia ajustado por integrantes		
Enfermera materno infantil	0,003	0,045
Laboratorio	0,80	0,84
Medicina familiar (no hipertensión arterial)	1,09	1,64
Medicina preventiva	0,61	0,77
Nutrición	0,33	0,50
Odontología	0,45	0.75
Planificación familiar	0,08	0.46
Imagenología	0,28	0,34
Trabajo social	0,36	0,37
Urgencias	0,26	0,57
Medicina familiar (por hipertensión arterial) ^a^	10,37	4,31

DE: desviación estándar

a Ajustado por el número de integrantes con hipertensión arterial en la familia

## DISCUSIÓN

El promedio de integrantes de la familia con hipertensión arterial fue de 3,05; la mayoría se encontraban en fase de jubilación y muerte, eran familias funcionales, el promedio de años con hipertensión fue de 11,65 y el promedio de integrantes con hipertensión fue 1,28. Determinar estas características desde la perspectiva de la familia, permitió identificar el contexto en el cual la hipertensión arterial sistémica puede ser tratada integralmente ^8^ ^,^ ^11^. 

La hipertensión arterial es una patología que aparece habitualmente a partir de la quinta década de vida, no obstante, cuando el abordaje se realiza desde la perspectiva de la familia, corresponde a la cuarta década. Esto probablemente se deba a que al calcular la edad familiar se incluye la edad de los hijos, lo cual necesariamente disminuye el promedio de edad; esta sería una característica de la familia con hipertensión arterial.

Está demostrado que la educación es parte del tratamiento no medicamentoso de la hipertensión arterial ^17^, pero para lograr el objetivo deseado, los contenidos deben diseñarse de acuerdo con las características de la población. En la familia con hipertensión arterial predomina la escolaridad secundaria, condición que debe valorarse al momento de definir los contenidos educativos y las técnicas didácticas, integrando así grupos de familias con hipertensión por nivel de escolaridad, escenario que probablemente sea más efectivo. 

Se encontró que la mayoría de las familias eran funcionales, característica que puede favorecer el control de la hipertensión arterial. Esto debido a que, en teoría, en una familia funcional los integrantes se procuran, reconocen y adoptan las necesidades individuales, facilitando así el logro de objetivos comunes, sin embargo, esta hipótesis debe ser comprobada en estudios posteriores ^1^ ^,^ ^9^.

La etapa del ciclo vital familiar en ocho de cada diez familias fue la de independencia y retiro, lo que significa que los hijos han empezado a salir del núcleo familiar para construir otra familia. El análisis de esta información en conjunto con la edad de la familia sugiere que las familias estudiadas iniciaron la integración como familia a temprana edad, por lo que será necesario comparar esta característica con la de la familia con diabetes o la familia con cualquier otra patología^ (^ ^1^ ^,^ ^5^.

Nuestros resultados muestran el uso constante de servicios de salud para el monitoreo de la hipertensión arterial, esto ha sido descrito en la literatura a nivel individual como un patrón de uso superior a lo reportado para otras patologías. A pesar de que la literatura evalúa principalmente el ámbito individual y, considerando que el propósito de nuestro estudio es evaluar a la familia con hipertensión, se podría extrapolar la conducta individual al ámbito familiar. 

No obstante, también se observó bajo uso de servicios preventivos, lo cual no es una condición ideal. En particular, las actividades de prevención con respecto a la hipertensión arterial son fundamentales para prevenir complicaciones crónicas, lo cual representa un área de oportunidad para proponer programas de salud ^12^.

La obesidad en el individuo es un serio problema de salud pública, en las familias con hipertensión arterial no es diferente. Cerca de la mitad de las familias presentaron obesidad, prevalencia inferior a la reportada para población mexicana ^18^, probablemente debido a la agrupación de varios individuos con obesidad en el mismo núcleo familiar. 

La alta prevalencia de obesidad sugiere la presencia de inadecuadas prácticas nutricionales y físicas que favorecen la presencia de complicaciones crónicas propias de la hipertensión arterial; sin embargo, la prevalencia de complicaciones crónicas en la familia con hipertensión es inferior a lo reportado para la población general, muy probablemente por la agrupación que se hace cuando la unidad de análisis es la familia ^19^. Se sugiere interpretar este escenario con precaución al momento de comparar prevalencias entre individuos y familias.

Una limitación de este estudio es la no identificación de las cifras de tensión arterial, ni el tipo de tratamiento medicamentoso. Además, se debe tener en cuenta que la población estudiada corresponde a una localidad de mediano tamaño, lo cual puede implicar que el perfil no necesariamente sea el mismo para familias con hipertensión que habitan en ciudades grandes, esta comparación se podría explorar mediante estudios similares. Finalmente, se deben señalar las implicaciones del muestro no probabilístico, entre ellas la limitación de extrapolación de datos a otras poblaciones.

En conclusión, la familia con hipertensión arterial es funcional, no obstante la mayoría se encuentra etapa de jubilación y muerte. Identificar el perfil epidemiológico de la familia con hipertensión arterial sistémica permite reconocer áreas de oportunidad para la implementación de políticas públicas orientadas al control de la hipertensión.
